# Identification and response to a putatively hypervirulent, carbapenem-resistant Klebsiella pneumoniae ST395 hospital outbreak in the UK

**DOI:** 10.1099/mgen.0.001571

**Published:** 2025-11-20

**Authors:** Mark I. Garvey, Robert A. Moran, Ana D. Sanches Ferreira, Jean Harker, Sarah Wilkes, Elisabeth Holden, Alan McNally

**Affiliations:** 1 Institute of Microbiology and Infection, College of Medicine and Health, University of Birmingham, Edgbaston, Birmingham B15 2TT, UK; 2 University Hospitals Birmingham NHS Foundation Trust, Queen Elizabeth Hospital Birmingham, Edgbaston, Birmingham B15 2WB, UK

**Keywords:** hypervirulent, carbapenem-resistant Klebsiella pneumoniae (hv-CRKP), infection prevention and control (IPC), nanopore sequencing

## Abstract

Here, we describe a putatively hypervirulent, carbapenem-resistant *Klebsiella pneumoniae* (hv-CRKP) outbreak. Nanopore sequencing guided the infection prevention and control (IPC) responses during the outbreak. Twelve patients were identified as either being colonized and/or having clinical infections with the putative hv-CRKP, with an all-cause mortality within 6 months of 36%. Multiple IPC responses were put in place, with enhanced cleaning of the environment being the key intervention to halt the outbreak. All isolates were ST395 and contained at least one *bla*_NDM_ carbapenemase gene, along with a variable array of other antimicrobial resistance and virulence genes, due to a rapidly evolving set of plasmids.

**Impact Statement**

The emergence of hypervirulent, carbapenem-resistant *Klebsiella pneumoniae* (hv-CRKP) poses a significant challenge to public health, especially in hospital settings where the risk of transmission is high. This study marks, to our knowledge, the first outbreak of a putative hv-CRKP in the UK, where nanopore sequencing provided critical insights into the pathogen’s genome, enabling rapid identification and precise tracking of the outbreak. The findings underscore the important role of genomic surveillance in enhancing infection prevention and control strategies. The outbreak was effectively mitigated through targeted interventions, demonstrating the practical utility of real-time genome sequencing in outbreak management.

## Data Summary

Illumina short-read sequences and Nanopore long-read sequences are available and have been deposited in NCBI under the BioProject accession number PRJNA1251496. Individual accession numbers are provided in Table S1.

## Introduction

*Klebsiella pneumoniae* is an opportunistic pathogen in the family *Enterobacterales*. Primarily a resident of the mammalian gastrointestinal tract, *K. pneumoniae* infections can span diverse body sites and include pneumonia, urinary tract infections, soft tissue infections and bacteraemia. Over the past two decades, there have been increasing reports of hypervirulent *K. pneumoniae* (hvKP) strains that exhibit enhanced virulence phenotypes, more readily causing infections, most strikingly in non-immunocompromised patients [1]. *K. pneumoniae* strains that display resistance to antibiotics have been reported worldwide, with multi- and extensively resistant phenotypes severely limiting therapeutic options. However, until recently, hvKP strains were generally antibiotic sensitive, while multiple antibiotic-resistant strains were generally not hypervirulent [1]. The convergence of hypervirulent and antibiotic-resistant phenotypes has been observed in multiple *K. pneumoniae* lineages in recent years, and this phenomenon has largely been driven by the acquisition of plasmids carrying antibiotic resistance genes, virulence genes or both [2].

University Hospitals Birmingham (UHB) is one of the largest UK hospital trusts, with ~3,000 beds, spanning four sites that cover the majority of the Birmingham population. The outbreak described here occurred at the Heartlands Hospital site Birmingham Heartlands Hospital site(BHH), which is a ~700-bedded hospital serving the southeast of Birmingham. BHH provides acute and general medicine, and other specialist services. The outbreak occurred in spring 2024 on a BHH vascular ward. The vascular ward contains 33 beds, with one bay of four beds, four bays with six beds and five single-bed side rooms. There are no ensuite facilities on the ward.

## Methods

Isolates were obtained from patient clinical or screening samples by plating on CARBA SMART agar (BioMérieux), which is selective for carbapenemase-producing *Enterobacterales* (CPE). Carbapenemase production was confirmed using the PCR-based Carba-R assay (Cepheid GeneXpert), as per UK Standards for Microbiology Investigations.

For long-read sequencing, strains were grown in Luria broth at 37 °C with 180 r.p.m. shaking overnight. Genomic DNA was extracted using the Monarch Genomic DNA Purification Kit (New England Biolabs) and quantified with the Qubit Broad Range dsDNA Kit (Thermo Fisher). Sequencing libraries were prepared using the SQK-NBD114 Barcoding Kit (Oxford Nanopore Technologies). Libraries were sequenced on the Oxford Nanopore Technologies GridION platform using R10.4.1 flow cells. Short-read sequencing (paired 250 bp reads) was performed on the Illumina HiSeq platform at MicrobesNG (Birmingham, UK). Genome assembly was performed using hybracter [3]. At the time of the outbreak, Oxford Nanopore reads were used alone and were later combined with Illumina data to generate hybrid assemblies.

Genomes were characterized using Kleborate [4]. F-type plasmid replicons were sub-typed using PubMLST (https://pubmlst.org/bigsdb?db=pubmlst_plasmid_seqdef). Insertion sequences were identified using ISfinder [5]. Plasmids were annotated manually and visualized using Gene Construction Kit (Textco Biosoftware).

A phylogeny was generated using isolate 0326575 as the reference to create a core SNP alignment with Snippy v4.4.5. This core SNP alignment served as input for Gubbins [6] to produce a recombination-free phylogeny. For the global context phylogeny, a reference-free core genome alignment was generated using Panaroo v1.5.2 [7]. Annotation of genomes was performed using Bakta v1.8.2 with the Bakta Light database v5.1 [8]. The core genome alignment was then used to construct an initial maximum-likelihood tree with RAxML v8.2.12 [9]. The final maximum-likelihood phylogenetic tree for the global context of ST395 *K. pneumoniae* isolates was generated using ClonalFrameML v1.13 [10] to account for recombination events. Additional genomes for the global context phylogeny were obtained from Pathogenwatch by selecting all available ST395 *K. pneumoniae* genomes (Table S1, available in the online Supplementary Material).

SNP distances were determined using hybrid-assembled genome sequences, with the genome of isolate 0326575 serving as a reference. Snippy v4.4.5 (https://github.com/tseemann/snippy) was used with the ‘--contigs’ flag to align assemblies to the reference and generate a core genome alignment. Gubbins was used to extract polymorphic sites and exclude sites predicted to occur via recombination [6]. SNP distances were calculated from the Gubbins-filtered polymorphic sites file using SNP-dists v0.6.3 (https://github.com/tseemann/snp-dists).

### Initial detection of carbapenem-resistant *K. pneumoniae*

The Infection Prevention Control team at UHB were alerted to the isolation of a carbapenem-resistant *K. pneumoniae* carrying *bla*_NDM_ in the clinical microbiology laboratory, from the urine sample of a patient (hereafter patient 3) on the ward with symptomatic (UTI)urinary tract infection. The isolation of carbapenem-resistant *K. pneumoniae* from this patient 3’s sample was confirmed on the Monday of outbreak week 1, which was the 23^rd^ day of patient 3’s admission.

As part of a newly implemented collaborative surveillance programme targeting CPE, the isolate was sent to the University of Birmingham for genome sequencing on the Oxford Nanopore GridION sequencing platform. Sequencing results were returned by the end of outbreak week 1. Sequence data revealed that the isolate was *K. pneumoniae* ST395. The isolate contained the extended-spectrum beta-lactamase gene *bla*_CTX-M-15_ and the carbapenemase gene *bla*_NDM-1_. Further interrogation of the genome using Kleborate [4] revealed that the isolate had a virulence score of 4 due to the presence of genes encoding aerobactin and yersiniabactin (Table S2). ST395 hypervirulent, carbapenem-resistant *K. pneumoniae* (hv-CRKP) has been reported in Germany [11] and in Ukraine [12]; however, this is the first time an outbreak has been reported in the UK.

### Genomic surveillance and outbreak identification

At this stage, patient 3 was isolated, and all beds and corresponding toilets on the ward were cleaned according to existing hospital protocols. A decision was made to immediately screen all patients on the ward, despite there being only one case on the ward at the time. This round of CPE screening took place in outbreak week 1 and yielded two further carbapenem-resistant *K. pneumoniae* isolates from rectal swabs obtained from patient 4 (isolate 326575) and patient 5 (326580), both of whom shared a bay with patient 3. Isolates 326575 and 326580 were subjected to long-read genome sequencing at the University of Birmingham, which confirmed that they were also *K. pneumoniae* ST395.

Following identification of *K. pneumoniae* ST395 in surveillance samples from patients 4 and 5, the bay they occupied was closed and emptied for deep cleaning using a sodium dichloroisocyanurate preparation, as described previously [13]. From this point, daily infection prevention meetings were convened to discuss the outbreak. Multiple infection-prevention interventions were initiated to limit the outbreak, including admission and weekly CPE screening, handhygiene education for both staff and patients, environmental microbiological sampling and enhanced cleaning of shared medical equipment for the period of increased incidence of CPE.

**Fig. 1.**
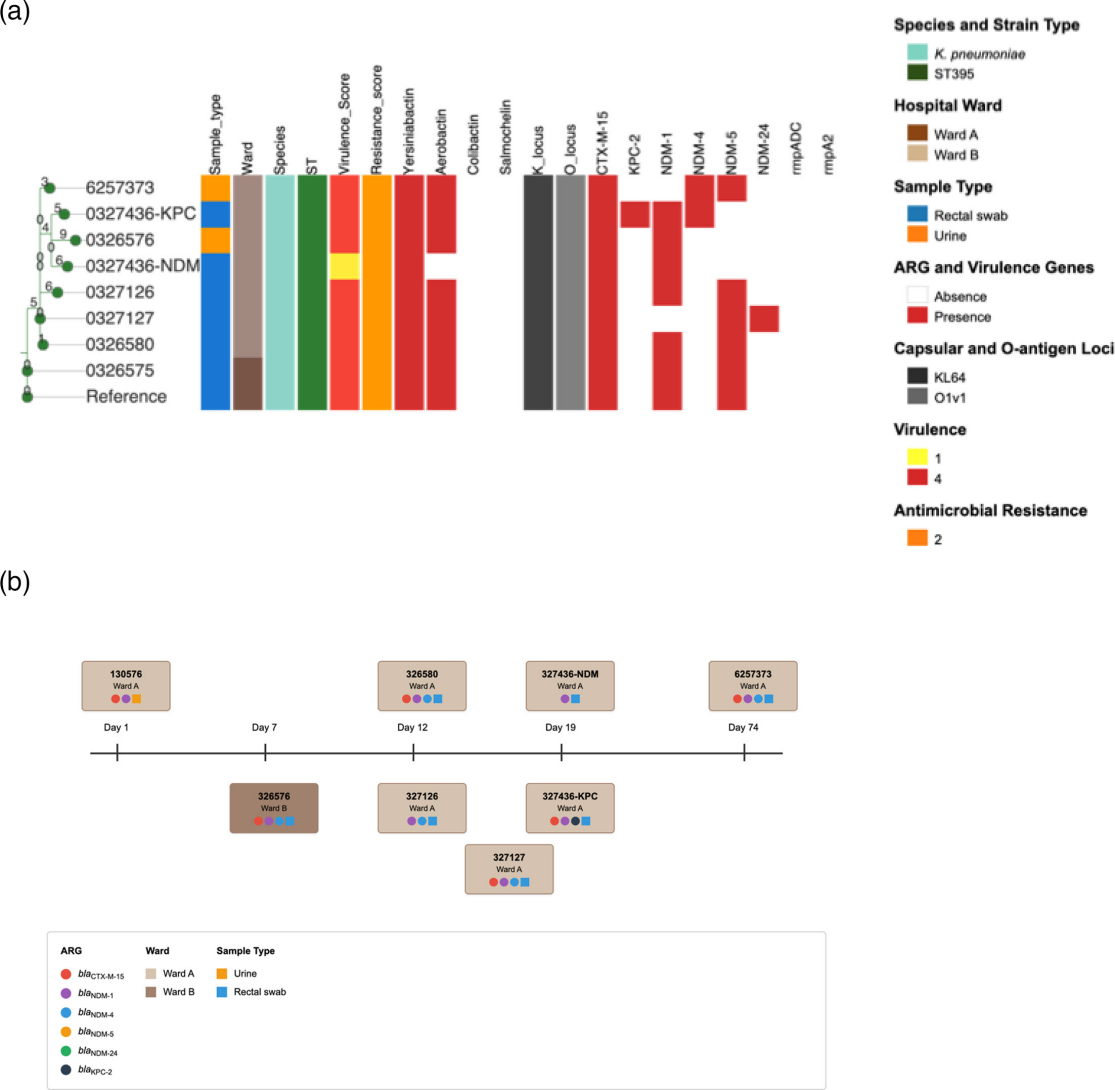
(**a**) Recombination-free maximum-likelihood phylogenetic tree showing the presence (red) or absence (white) of virulence loci (yersiniabactin, colibactin, aerobactin*,* salmochelin and *rmp* genes) and acquired resistance genes (including ESBLs *bla*_CTX-M-15_ and carbapenemases *bla*_KPC_ and *bla*_NDM_ genes). The isolates all show KL64 and O1v1. The numbers indicate branch lengths in the number of point mutations. Epidemiological metadata – sample type and ward – are shown alongside the genomic features of the isolates. (**b**) Temporal distribution of the isolates across hospital wards, including sample type and antibiotic resistance genes.

Screening in week 2 of the outbreak yielded carbapenem-resistant *K. pneumoniae* from a further four patients on the ward: patient 6 (isolate 327126), patient 7 (327127), patient 8 (6257373) and patient 9 (327436-NDM and 327436-KPC). Long-read sequencing at the University of Birmingham confirmed that these were also *K. pneumoniae* ST395. The identification of these additional cases prompted closure of the entire ward to facilitate deep cleaning of all bays and side rooms, replacement and installation of a new toilet on the ward, and replacement of all mattresses, curtains and pillows throughout the ward. The ward was partially reopened 10 days after closure. A timeline of this sampling is represented in Fig. 1.

### Retrospective surveillance and index patient identification

As part of the outbreak management and investigation process, three additional cases (patients 10–12) were identified on other wards across the Trust through contact tracing of patients who had previously been on the vascular ward. An extensive retrospective investigation of patients who had spent time on the vascular ward identified two cases from prior to outbreak week 1: patient 1 and patient 2. Patient 1 had spent time on the outbreak ward in October 2023, and patient 2 (unrelated to patient 1) yielded a clinical specimen in January 2024, having spent 17 days on the ward. Patient 2 was found to be a sibling of patient 3, who was the source of the clinical isolate (326576) that triggered this investigation.

Although isolates from patients 1–2 and 10–12 were not subjected to genome sequencing as part of this study, typing at the Antimicrobial Resistance and Healthcare Associated Infections (ARMHAI) reference laboratory at the UK Health Security Agency (UKHSA), Colindale, revealed that they were all *K. pneumoniae* ST395.

In total, we believe we identified 12 cases of this putative hv-CRKP. The patients’ ages ranged from 51 to 88, with an average age of 71. Ten of the patients were male and two were female. Three patients died within 3 months of having a positive screen/clinical specimen with hv-CRKP.

### Context and features of outbreak strain genomes

**Fig. 2.**
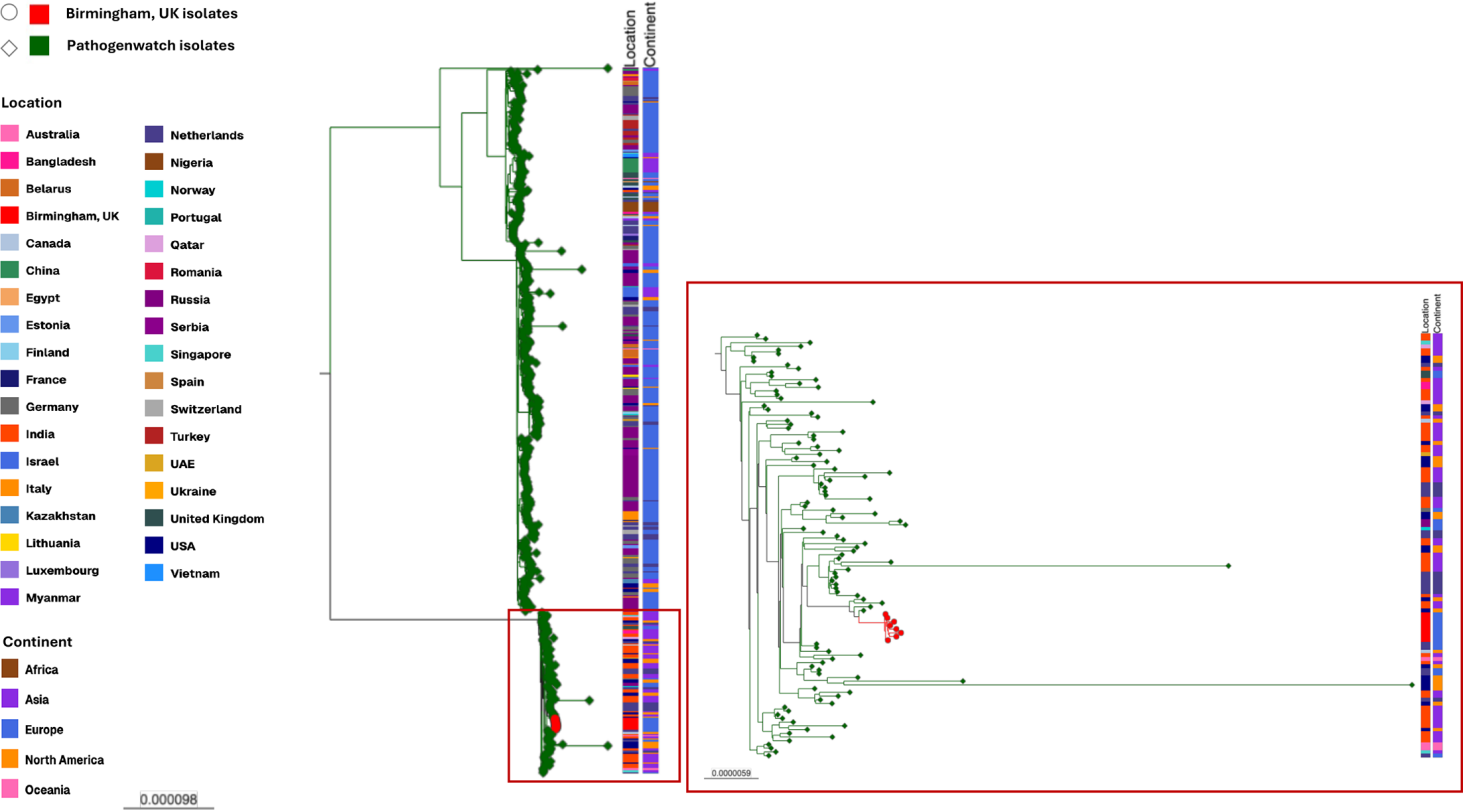
Recombination-free maximum-likelihood phylogenetic tree of the Birmingham ST395 isolates (red circular tree tips) versus a global selection of ST395 isolates retrieved from Pathogenwatch (listed in Table S1). The clade of the phylogeny containing the Birmingham strains is magnified to show they form a distinct monophyletic cluster. The tree is annotated by country and continent of origin of each strain.

To confirm the extent to which these strains were related, we also sequenced them on the Illumina NextSeq platform to perform definitive SNP analysis, which confirmed all isolates to be within 12 core-genome SNPs from each other, indicative of likely transmission (Fig. 1). The Birmingham ST395 isolates were contextualized using ST395 *K. pneumoniae* genomes from Pathogenwatch. These genomes included isolates from five different continents (Fig. 2). The phylogenetic analysis revealed that the ST395 isolates from this study formed a small and clear monophyletic cluster, consistent with local clonal expansion rather than separate introductions into this hospital. The isolates from this study formed part of a larger cluster that included isolates from Asia, North America and Oceania, indicating that they were more closely related to these isolates than to European isolates, which formed a separate cluster in the phylogeny (Fig. 2). The diversity in resistance and virulence gene carriage in the Birmingham isolates was caused by a surprising level of plasmid diversity observed in each isolate (Fig. 1). The isolate genomes comprised an ~5.5 Mb circular chromosome and anywhere between two and six circular plasmids per genome.

**Fig. 3.**
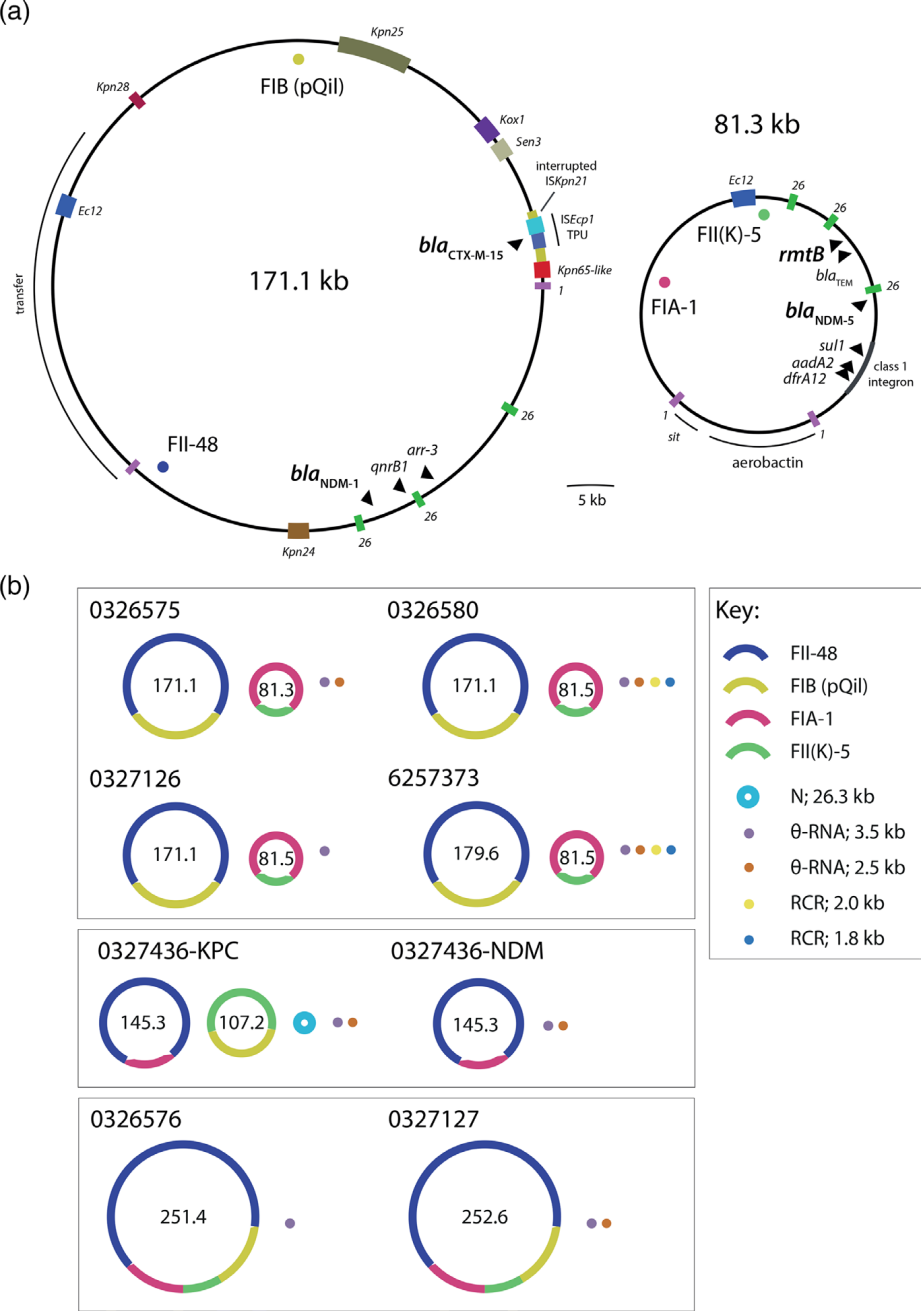
Plasmid content variation in outbreak isolates. (**a**) Circular, scaled maps of the F-type plasmids in index isolate 0326575. The locations of plasmid replicons are indicated by labelled dots; antibiotic resistance genes by labelled triangles; insertion sequences by labelled boxes (e.g. ‘26’ for IS26); and the transfer region, aerobactin and sit siderophore determinants by labelled arcs. (**b**) Overview of plasmids in each isolate’s genome, grouped according to the configuration of F-type plasmids. The sizes of F-type plasmids are shown inside the circles that represent them, while the replicon types and sizes of other plasmids are listed in the key. Plasmids in this figure are not drawn to scale, and the extents of the coloured lines in the F-type plasmids are not representative of sequence features.

The most common configuration of plasmids in these genomes featured two large F-type plasmids. Representative of this, isolate 0326575 contained four plasmids with sizes of 171.1, 81.3, 3.5 and 2.5 kb. In this configuration, the 171.1 kb F-type plasmid contained FII-48 and FIB(pQil) replicons, while the 81.3 kb F-type plasmid contained FII(K)−5 and FIA-1 (Fig. 3a). This configuration was present in a further three isolates, while others featured different configurations of the F-type elements and their accessory content (Fig. 3b). In two isolates, all four F-type replicons were present in a single 251.4 or 252.6 kb cointegrate. The formation of these cointegrates appears to have been mediated by IS26, which is consistent with the extensive body of experimental evidence for this element [14]. In the remaining two isolates, the FII-48 and FIA-1 replicons were located on a 145.3 kb plasmid, and in one isolate, the FII(K)−5 and FIB(pQil) replicons were located on a 107.2 kb plasmid (Fig. 3b). One of the isolates, 0327436-NDM, did not contain this 107.2 kb plasmid, lacking the FII(K)−5 and FIB(pQil) replicons, along with the aerobactin operon it contained, accounting for this isolate’s reduced Kleborate virulence score of 1 (Fig. 1). Notably, isolate 0327436-KPC carried an additional *bla*_KPC-2_ carbapenemase gene on a 26.3 kb N-type plasmid, which is a deletion derivative of the pQEB1 lineage that has been circulating in Birmingham [15].

### Infection prevention and control measures

As soon as the real-time genomic data identified an outbreak, multiple infection control interventions were put in place to limit the outbreak, including admission and weekly CPE screening; hand hygiene education for both staff and patients; ward closure to undertake enhanced cleaning of the bays and side rooms, each time with new air mattresses and curtains introduced, as described previously by Garvey *et al*. [13]; enhanced cleaning of shared medical equipment; decontamination of the sink traps with peracetic acid; and estates works such as replacement of the toilets on the ward, to name but a few interventions. Only after confirmation that the outbreak strain was isolated in the ward in July could we enforce ward closure, enabling an enhanced cleaning of the ward environment, decontamination of the sink traps and replacement of the ward’s broken toilet, resulting in no further cases of the putative hv-CRKP on the ward.

### Conclusion

Rapid sequencing of outbreak strains has transformed our understanding of the phylogeny and epidemiology of pathogens. In the UK, whole-genome sequencing is not routinely used to differentiate CPE healthcare-associated strains or strains involved in an outbreak. Using conventional laboratory methods, the identification of the putative hv-CRKP would not have occurred. Whilst the 6-month all-cause mortality of the infected patients was 36%, this is not sufficient to clearly delineate the strain as truly hypervirulent, despite the genomic signatures of the strain. Nanopore sequencing focused our infection prevention and control (IPC) strategy on environmental cleaning and estates work, by enabling us to engage with clinicians and hospital management. Our experience adds to the growing body of evidence that real-time long-read sequencing holds great promise for making an important contribution to outbreak investigations and better targeting of IPC interventions to reduce the burden of infectious disease.

## Supplementary material

10.1099/mgen.0.001571 Uncited Supplementary Material 1.

## Abbreviations

ARMHAI: Antimicrobial Resistance and Healthcare Associated Infections
CPE: carbapenemase-producing *Enterobacterales*
hv-CRKP: hypervirulent, carbapenem-resistant *Klebsiella pneumoniae*
hvKP: hypervirulent *Klebsiella pneumoniae*
IPC: infection prevention and control
UHB: University Hospitals Birmingham
UKHSA: UK Health Security Agency
